# A Qualitative Analysis of Perspectives on Self-directed Violence in a Prospective Longitudinal Study of Young Women With and Without Childhood ADHD

**DOI:** 10.1177/10870547231221729

**Published:** 2024-01-31

**Authors:** Shaikh I. Ahmad, Stephen P. Hinshaw

**Affiliations:** 1 University of California, San Francisco, USA; 2 University of California, Berkeley, USA

**Keywords:** Non-suicidal self-injury, self-harm, qualitative method, ADHD, longitudinal

## Abstract

**Objective:**

Non-suicidal self-directed violence (NSSDV) is a significant and growing youth public health crisis. Girls with ADHD are at increased risk of engaging in NSSDV, yet qualitative studies with this population—to better understand manifestations, motivations, and developmental course—are lacking.

**Method:**

We conducted semi-structured, qualitative interviews with a sub-sample of 57 young women (32 with childhood ADHD, 25 neurotypical comparisons; mean age of 27 years, part of a larger prospective longitudinal study) regarding histories and manifestations of NSSDV.

**Results:**

Inductive and deductive analysis revealed several key themes, including self-perceived reasons for engaging in NSSDV (affect regulation, attention seeking, self-punishment, asserting control), impulsivity, secretiveness, and in some cases motivations for desistance.

**Conclusion:**

Findings underscore the importance of early education and screening, especially among high-risk clinical populations. Increased resources and supports for professionals, parents, and peers are indicated, along with countering the persistent stigma associated with ADHD and NSSDV.

Self-directed violence (SDV), as defined by the Centers for Disease Control and Prevention (Crosby et al., 2011), comprises both suicidal self-directed violence (in which the intention is to end one’s life) and non-suicidal self-directed violence (NSSDV)—also referred to as non-suicidal self-injury (NSSI)—which encompasses actions such as cutting, burning, or hitting oneself without the express intention of dying. NSSDV is an issue of major clinical and public-health concern, given its early age of onset (often starting in late childhood or early adolescence), high prevalence rates, and significant predictive power for later suicidal behavior (Ammerman et al., 2018; Brager-Larsen et al., 2022; Muehlenkamp et al., 2012; Swannell et al., 2014). Herein, we focus on personal accounts of prior engagement of NSSDV via a qualitative investigation featuring young women with a childhood history of attention-deficit hyperactivity disorder (ADHD)—a known risk factor for NSSDV (see Hinshaw et al., 2022)—plus a neurotypical comparison group. Two understudied aspects of SDV include (a) the contexts within which youth engage in such behaviors (i.e., situational and environmental factors that may influence an individual to engage in such behavior), and importantly (b) its developmental course (i.e., factors that affect both persistence and desistance). These two topics are the focus of the present study.

## Brief Background on NSSDV

NSSDV is a complex, heterotypically continuous phenomenon (Prinstein, 2008). Although it often starts in adolescence, it can emerge as early as childhood. Prevalence estimates vary from 17% to 18% within non-clinical adolescent samples to rates of over 50% among clinical groups (Muehlenkamp et al., 2012; Swannell et al., 2014; Westers & Culyba, 2018). Developmental pathways leading to child and adolescent NSSDV are likely to be both equifinal and complex. One clear risk factor is childhood manifestations of psychopathology, including a range of internalizing and externalizing behaviors as well as ADHD (Allely, 2014; Fox et al., 2015; Meza, Owens, & Hinshaw, 2021; Swanson, Owens, & Hinshaw, 2014). Additional risk factors for NSSDV are especially relevant for youth with ADHD, including impulsivity, comorbidity with depression and anxiety, poor executive functioning, and significant risk for social and interpersonal difficulties (Meza, Owens, & Hinshaw, 2016; Swanson et al., 2014).

Several core reasons (or motivations) for engaging in NSSDV have been suggested (Edmondson et al., 2016; Klonsky, 2007). These include affect regulation, anti-dissociation (to feel physical pain as opposed to emotional pain), personal agency (asserting control over one’s body), avoidance of making a suicide attempt, interpersonal influence (seeking attention or help from—or attempting to manipulate—others), self-punishment, and sensation seeking, to name the most salient. Broadly, these reasons/functions fall into two main categories: interpersonal and intrapersonal (Gardner et al., 2021). There is also growing evidence that most individuals engage in NSSDV for multiple reasons (Victor et al., 2016). Some appear to do so for positively reinforcing reasons (i.e., because they experience a physiological arousal or “rush” when engaging in such behaviors) whereas others do so for negatively reinforcing reasons (i.e., experiential avoidance; Chapman et al., 2006; Nock, 2010). Especially relevant for adolescents, some NSSDV behaviors might be modeled and influenced by peers, at least initially (Jarvi et al., 2013; Prinstein et al., 2010), as a means of gaining attention or acceptance. Such interpersonal modeling might be more relevant for girls than boys (Prinstein et al., 2010). A robust literature also exists regarding the relation between impulsivity and NSSDV, suggesting that emotion-related impulsivity might play an important role (Lockwood et al., 2017). Few longitudinal studies have examined such reasons for NSSDV, although affect regulation is commonly identified (Taylor et al., 2018).

## NSSDV in Context

A growing body of research suggests various proximal factors that may influence engagement in NSSDV (see Rodríguez-Blanco et al., 2018; Wilkinson et al., 2011). Yet Likert-scale based measures and questionnaires may not adequately capture important contextual and environmental factors. More recently, qualitative and micro-longitudinal studies—such as ecological momentary assessments (EMA) and daily diaries—have shed some light on this subject. Such research highlights the important roles of interpersonal stress, emotion dysregulation, impulsivity, rumination, and lack of social support as proximal risk factors for NSSDV (Rodríguez-Blanco et al., 2018). Still, additional contextual information is needed in identifying more targeted intervention strategies as well as more broad prevention efforts.

## Developmental Course of NSSDV

Importantly, there is a paucity of information regarding the developmental course of NSSDV (Cipriano et al., 2017; Taliaferro et al., 2019). That is, once individuals have initiated such behavior, identifying the environmental and inter-/intra-personal factors that contribute to its continuation/maintenance or desistance is a priority (for data on persistence following adolescence in females with ADHD, see Meza et al., 2021). Better understanding of the multiple factors influencing the initiation, maintenance of, and desistance from SDV could elucidate and inform targeted prevention and early intervention strategies. For example, it is entirely possible that the reasons youth initiate NSSDV behavior may be different from reasons for the persistence of such behaviors (Gardner et al., 2021; Taliaferro et al., 2019).

## Benefits of Qualitative Research on NSSDV

Crucial to enhanced understanding of NSSDV are (a) knowledge of transactions between individuals and their environments, (b) thorough examination of intrapersonal and interpersonal events that precede such behaviors, and (c) comprehension of how changes in affect are associated with NSSDV. Yet many of these processes are difficult to assess via self-report or rating scales, given the restrictive nature of such measures. Qualitative research has been used in the social sciences, including research with clinical populations, to gain a deeper understanding of environmental, contextual, and individual factors that can then be translated into clinical strategies for the alleviation of human suffering (Kleinman, 1988; Lasky et al., 2016; Weisner et al., 2018). Such investigations in youth are rare for NSSDV (e.g., Biddle et al., 2013; Sinclair & Green, 2005; Wadman et al., 2018). Furthermore, much current research focuses on individuals at the extreme end of the clinical range (e.g., individuals in a psychiatric inpatient unit), which may not capture sufficient experiences along the continuum of NSSDV.

## Present Study

Leveraging a diverse, prospective longitudinal sample of young women with and without childhood-diagnosed ADHD (see Owens, Zalecki, Gillette, & Hinshaw, 2017), we employed a semi-structured, qualitative interview approach to examine: (a) reasons why youth engage in NSSDV, (b) contextual factors (both intra- and inter-personal) related to such behaviors, and (c) developmental course (including onset, maintenance, and desistance). We are not aware of any qualitative SDV-related studies on youth with ADHD that leverage prospective longitudinal samples. The current investigation is well-positioned to answer these questions, given our previous findings of higher rates of SDV behavior within this sample (Hinshaw et al., 2012; Meza et al., 2021).

Utilizing a thematic analysis approach, we had the following aims. First, using deductive thematic analysis, we explored several *a priori* themes related to NSSDV based on extant theory and research. Two key themes included (a) reasons why youth engage in NSSDV (Theme 1) and (b) the influence of others on youth NSSDV (Theme 2). Second, using inductive thematic analysis, we examined participant interviews for novel themes and subthemes (i.e., those not necessarily informed by extant research) that appear pertinent to NSSDV-related behavior.

## Method

### Participants and Procedure

We used prospective data from the Berkeley Girls with ADHD Longitudinal Study (BGALS), the largest and longest-running prospective study, to our knowledge, of young women diagnosed with ADHD during childhood (Hinshaw, 2002; Owens et al., 2017). The overall sample consisted of 228 girls (140 diagnosed with ADHD and 88 age/demographically-matched neurotypical comparison girls) who have been followed via four waves of data collection for over 16 years (retention rates ranged from 92–95% across Waves 2–4). The mean age at Wave 1 was 9.6 years; Wave 2 = 14.2 years; Wave 3 = 19.6 years; and Wave 4 = 25.6 years; (Wave 2 not included in this investigation). The sample was racially and socioeconomically diverse and generally representative of the greater San Francisco Bay Area in terms of race, income, and parental education.

For the present study, we used a parallel approach to that of Weisner and colleagues, who also conducted qualitative interviews using a different prospective longitudinal sample (see Lasky et al., 2016; Weisner et al., 2018). Herein, between 1 and 4 years after their young-adult assessment (Wave 4), participants were invited to take part in an in-person, open-ended, qualitative interview (mean age: 27.7 years). For participant recruitment purposes, a 2 × 2 matrix was created based on two key dimensions of interest generated from our quantitative data: (a) those with childhood ADHD versus non-ADHD comparison peers, and (b) those who had previously reported engaging in SDV behavior (either suicidal or non-suicidal) versus those who had not. Given present aims, the sample was enriched in an unbalanced design for overinclusion of (a) individuals with ADHD and (b) those who previously reported SDV. In total, 81 participants were contacted. Of those, 24 were unable to be part of an in-person interview (e.g., no longer living in the area). A total of 57 participants completed qualitative interviews. Table 1 presents demographic information.

**Table 1.** Participant Demographic Information.

	W1 diagnostic status	
	ADHD *n* = 32	Comp *n* = 25
Age (years)	27.8 (2.0)	27.5 (1.6)
Education^a^ (%)		
High school diploma/GED	20 (63)	5 (20)
“Associate’s degree”	1 (3)	1 (4)
Trade program/certificate	4 (12)	1 (4)
“Bachelor’s degree”	5 (16)	16 (64)
‘‘Master’s degree’’	2 (6)	2 (8)
Race/ethnicity (%)		
White—non Hispanic	22 (69)	12 (48)
African American	5 (16)	5 (20)
Hispanic/Latina	3 (9)	3 (12)
Asian American	1 (3)	5 (20)
Native American	1 (3)	0 (0)
Self-directed violence (%)		
No self-directed violence	14 (44)	18 (72)
NSSDV only	11 (34)	5 (20)
Suicide attempt only	1 (3)	0 (0)
NSSDV and suicide attempt	6 (19)	2 (8)

*Note*. ADHD = ADHD group; Comp = non-ADHD/neurotypical comparison group.

a Highest level of education as of the W4 follow-up visit.

Qualitative interviews averaged 2 hours and covered a range of topics (e.g., family, peers, relationships, academics, and employment, in addition to SDV). Interviewers included the first author plus two post-baccalaureate research assistants (RA), each of whom received over 40 hours of training, including practice interviews, shadowing, direct observation, and feedback. The portion of the interview pertinent to the present study focused on questions related to SDV–both suicidal behavior and NSSDV. Given the lower frequency of suicidal behavior compared to NSSDV in the sample, we focus on NSSDV herein. All participants were asked about their thoughts on the topic (e.g., “Why do you think some people might want to hurt themselves on purpose”?). For those who endorsed engaging in prior NSSDV, additional follow-up questions were asked (refer to Supplemental Materials Section 1 for follow-up prompts). Through these procedures, even those participants who had not reported prior engagement in NSSDV could discuss why they thought other people might engage in such behaviors. Study approval was provided by the University of California, Berkeley’s Committee for the Protection of Human Subjects.

### Measures—Quantitative Data

We used the following key measures from the four waves of existing quantitative data to build the aforementioned unbalanced 2 × 2 recruitment matrix.

*ADHD Diagnostic Status*: During Wave 1, parents were administered the Diagnostic Interview Schedule for Children, 4th ed. (DISC–IV). This well-validated structured clinical interview has been used widely for childhood diagnoses of psychopathology. For the present study, participants diagnosed with either ADHD–Inattentive (ADHD-I), or ADHD—Combined type (ADHD-C) were assigned to the ADHD group, otherwise to the non-ADHD Comparison group (see Hinshaw, 2002, for details).

*NSSDV*: Data on NSSDV were obtained at both Wave 3 and Wave 4. At Wave 3, participants were administered the Self-Injury Questionnaire (Claes et al., 2001; Hinshaw et al., 2012; Swanson et al., 2014), through which they were asked whether they had deliberately harmed themselves using various methods, also assessing frequency and severity. At Wave 4, NSSDV was assessed from the well-validated Self-Injurious Thoughts and Behaviors interview (SITBI; Nock et al., 2007). If, by Wave 4, the participant endorsed engagement in a more serious form of NSSDV (i.e., scratching until one bleeds, cutting, burning) at least once in their life, the respective variable was marked “yes”; otherwise, “no.”

### Qualitative Interview and Methodology

In this qualitative study we utilized an Ecocultural Family Interview approach (Weisner et al., 2018). Participants were asked open-ended questions on a range of topics—including SDV—and were encouraged to “tell their story” in their own words. Interviews were digitally audio-recorded with participant permission. Each interview was transcribed into Microsoft Word by a trained RA, then reviewed for quality by a second RA. Transcripts were then uploaded to an online qualitative analysis system (Dedoose; www.dedoose.com) to capture interview excerpts for indexing, grouping, coding, and analysis.

*Coding and analysis*: We created a coding manual in a two-step process, utilizing thematic analysis for qualitative interviews (Boyatzis, 2009; Braun & Clarke, 2006). Herein, we discuss only themes related to NSSDV. The coding manual was refined in an iterative fashion wherein it was tested on multiple transcripts and reviewed by a coding team composed of the first author and two additional, trained, post-baccalaureate RAs. Initial coding reliability was established during development of the coding manual, with kappa coefficients across all three coders >.80. Once the Dedoose system was operational with the finalized codes and all interview transcripts, inter-rater reliability was again assessed across all three coders, via a randomly selected subset of interview excerpts. Inter-rater reliability was again high, with kappa coefficients across all three reviewers >.85. Each interview was then coded by an RA and reviewed by the first author. Participant excerpts are presented below to elucidate common themes; numeric IDs are used to identify unique participants.

Each theme was established in one of two ways. First, we established four a priori themes based on existing research and theory (deductive thematic analysis). These themes—theoretically informed by extant research as well as gaps in current research—included the following: Theme 1—reasons for engaging in NSSDV (i.e., functions of NSSDV); Theme 2—whether NSSDV behavior was influenced by others; Theme 3—reasons for desisting from NSSDV; and Theme 4—NSSDV and impulsivity. Second, we employed an inductive thematic analysis approach, which entailed reviewing a subset of interview transcripts for additional themes, identifying Theme 5—keeping NSSDV a secret.

## Results

Refer to Table 1 for demographic information. Of the 57 study participants, 25 reported previously engaging in some form of SDV behavior, with 24 engaging in NSSDV (see Supplemental Materials Section 2 for additional details). Eighteen of these twenty-four had received a childhood ADHD diagnosis. Of the 24 participants who reported NSSDV, the vast majority (22) reported cutting themselves with a sharp object or scratching on purpose to the point of bleeding as their main form of NSSDV. Seventeen of these twenty-four reported engaging in NSSDV before high school; two reported starting in elementary school. All but one participant who endorsed NSSDV reported doing it repetitively. Most reported engaging in repetitive NSSDV for over a year; for some, as many as 8 years. Notably, only one individual (who had engaged in NSSDV only—that is, had not made a suicide attempt) reported having gone to the emergency department or being hospitalized for their NSSDV behavior.

### NSSDV Themes

#### Theme 1: Reasons for Engaging in NSSDV

The following reasons for engaging in NSSDV were identified: (1A) affect regulation, or a means of expressing of emotional pain; (1B) to gain attention from others; (1C) attempts to assert control over one’s life, and (1D) the belief that they deserved pain/punishment. Importantly, and as is evident in some excerpts below, all but three participants endorsed more than one reason for engaging in NSSDV.

*Sub-Theme 1A (affect regulation/expression)*: The most common reason for engaging in NSSDV was to help regulate emotions or to physically express emotional pain. The most common emotions discussed were loneliness, sadness, or feeling frustrated/overwhelmed. For example:

(ID 2): I did it like as a release. . . It was therapeutic in a scary way to me. Like you’d just start with a little cut and then you just keep going and going and it was just like a release. . . like, took your mind off of everything for a minute. You didn’t have to focus on the inside of your head. You could get out of your body, if that makes sense. I think I was literally just sitting in my room and like it was dark and quiet and I was like alone in my thoughts – and I just remember seeing scissors on my desk and schoolwork or something and I was like, ‘Those look good’. . .

Similarly:

(ID 1): When I was an adolescent through, like, teens, I did it because the physical pain was easier to deal with than the emotional. So like, if I felt like if I had a gash in my soul. . . it’s easier to deal with a gash on my arm. It’s physical, it hurts, it’s bleeding. I can take care of it. I can show somebody and be like, hey look what I did. ‘Cause part of it was for attention. That’s why I did it. I was like there’s something wrong with me inside. But I can’t explain what it is. So look at this physical thing on me. . . So I could register, so my body registered as something real. ‘Cause emotional pain was just too hard to deal with ‘cause I didn’t understand why I was feeling like that.

In short, participants described engaging in NSSDV to make sense of (or make visible) psychological distress, feeling that they could not communicate such to others. Indeed, most who endorsed this sub-theme said that they could not talk with anyone about these feelings. The NSSDV provided temporary relief or release, often described as a physiological response.

*Sub-Theme 1B (gain attention from others)*: Many individuals reported engaging in NSSDV, especially initially, to gain attention from others. Of note, all participants who endorsed this sub-theme also endorsed 1A (affect regulation). Some described this as a “cry for help,” usually directed at parents or authority figures:

(ID 4): I think I was doing very poorly in school and I think I was getting bad grades and was so overwhelmed with these projects. And I didn’t feel like my mom was listening to me and I just felt like I was so unhappy and no one was listening and I couldn’t. . . like I was starving for attention and I couldn’t get any attention, even though I always had a lot of attention, it was. . . a different kind of attention. . . I was getting this negative type of attention when I really needed—I don’t even know what kind of attention I needed, I just knew that I wasn’t getting the attention that I needed instead of the attention I was getting.

Others described the need for attention among peers, especially for those who had friends who also engaged in NSSDV.

(ID 5): I think after I did it once. . . Maybe because I got attention for it. . . and my friend was like ‘Oh, you did that, we need to go talk to somebody.’ And then everybody made a big deal about it. And then it became a thing. . . For most of the time that I was cutting—I didn’t get attention from teachers and my parents. I think the attention that I was getting was social so like from my friends. Like I said, I would cut and all my friends would cut and we would come together and be like ‘Oh sob story.’ It was like we—it almost became like a social thing. We would all kind of like bond over it. So it was almost like relief, the cutting became a relief through a social bonding.

*Sub-Theme 1C (assert control)*: Just under half of respondents discussed experiencing a lack of perceived control over their life or environment; engaging in NSSDV was an attempt to assert control over their body. For most, this desire was often borne from frustration:

(ID 6): I felt completely out of control of my whole life. It really started after my parents split up and. . . everything around me felt like falling apart. And so for me, it was like I can take all of this pain that I’m feeling and all of these uncontrollable, everything going on in the world, and I can channel it into something that I’m in control of. . . I know what’s going on that I can choose what I’m doing. . . especially because, you know, my mom would watch when I was taking my medication. Everything about my life felt dictated, and it was like, when I am in the shower, I can cut myself, and no one will know until afterward. That was the time that I could just do whatever I wanted to do. I mean it felt much more like, obviously it physically hurt, and then there was an element of. . . It felt good to sort of have some sort of an actual physical representation of, you know, sort of what’s going on inside. But partly for me, it just felt like a way to have control of something that was going on.

*Sub-Theme 1D (deserving pain/punishment)*: This sub-theme was more punitive in nature. Six individuals articulated a feeling of anger directed at themselves, related to frustration, a belief that something wrong with herself, or self-blame regarding life events:

(ID 6): It was more intending to harm myself instead of just cutting ‘cause it felt good. I was still cutting because it felt good, but part of the reason it felt good was because I was harming myself. . . Again, this is a little bit of the ADD thing, sort of having grown up thinking I’m different, I’m wrong, there is something wrong with me. Sort of having the feeling that I need to be fixed and hitting this point where I’m like, well what if I can’t be fixed? I’ve been dealing with this for how many years of my life. It’s not getting better. . . I would see myself, because my family was falling apart, I saw myself as the source of that, and I just looked at it and thought, ‘Well, look at all those bad stuff that I’m contributing to the world.’ I’m not even getting anything positive out of it. I’m not getting better and I’m still continuing.’ I have to do therapy and I have to do meds. . . So it’s kind of, what’s the point in continuing if no one is getting anything out of it. . . so at that point it really became more of, I want to hurt myself, to make myself feel like, I guess in a sense it was like a punishment sort of thing. So it’s like the world is falling apart around me; everyone hates me; I hate myself.

Similarly:

(ID 7): From the outside, a lot of people can’t understand, but there can be a lot of anger towards oneself when you have a diagnosis of a disorder. . . and you feel like it’s had a huge negative impact on your life. And so, you start hating yourself because of what’s happened to you because you think it’s your fault. . . And there’s this chronic background noise of unhappiness that just won’t go away. And sometimes. . . it almost feels like a punishment or it feels like a way of getting control of yourself. Or being suicidal because they just don’t want to have another day in this life where they—just having this life that you just don’t enjoy, or you just feel miserable all the time. . . What I recently realized is, it started back in elementary school. I would hit myself, and I didn’t realize until now that that was self-harm. And that was me acting out my own anger onto myself.

Importantly, for most of these individuals, NSSDV turned into a cycle. That is, events would trigger an emotional reaction leading to NSSDV (often with the recognition, in retrospect, that it did not help their situation), which then brought about additional emotions, triggering additional NSSDV. NSSDV thus became a negatively reinforcing pattern (e.g., trying to temporarily “escape” from negative feelings by harming themselves)—much like a stress response, for which NSSDV took on a feeling of a “habit,” “ritual,” or “compulsion” that was difficult to resist.

(ID 8): So my self-harming behavior started in middle school as attention-seeking behavior, which is often how it is. . . and then moved into something that was really cathartic. And that it was the only way that I could truly actually express what it felt like in the inside of my head, you know, was just to have it show on the outside. . . I cut and I scratched. . . After a certain point, it then would kind of became an anxiety response. You know like I would be in a situation that would be really difficult for me. And I would look down and realize I was like compulsively scratching my arm.

Similarly:

(ID 9): I think. . . honestly, this has been a recent discovery about myself. . . I chew my cheeks *a lot*. I chew them to the point of bleeding and stinging basically, so to the point that they hurt. And I’ve kind of realized that this is a reaction to when I’m stressed. Just being more aware of myself recently over the years, I’m realizing, ‘Okay, when you’re really stressed, you do inflict some kind of pain to yourself because you’re sitting here gouging out the inside of your cheeks and gums and stuff. . .’ I had never even thought of it until I paid attention to myself when I do when I’m stressed and everything. . . It almost becomes like OCD: I can’t stop.

#### Theme 2: NSSDV Influenced by Others

Just under half of respondents who engaged in NSSDV reported that their NSSDV was at least partially influenced by others—either peers, TV/film, or other media. Interestingly, three participants explicitly recalled seeing it for the first time, or getting the idea of NSSDV, from the movie *Thirteen*. As one participant noted:

(ID 10): “I went through the whole dark emo goth whatever phase, and I don’t know—just like one of my friends came over and she showed us, ‘Oh. . . look what I did.’ And. . . for whatever dumb reason we both tried it. And my other friend was like, ‘Oh this is stupid.’ And then that kinda sent me in a – just downward spiral I guess for the next couple years.”

Similarly:

(ID 8): So I had a couple [friends], like I think, my three closest, I would say—‘cause I wasn’t really close with anyone. But the three girls that I hung out with the most were all really into self-harm. . . I can’t remember where I learned about it. It was just one of those things where, it was just a thing. And it was an interesting thing to be in the middle of, to look at these three girls and know definitively or think that I knew definitively that all three of them were doing it to seek attention. And I was doing it because I was hurting.

#### Theme 3: Reasons for Desisting NSSDV Behavior

When individuals were queried about reasons for desisting from NSSDV, three patterns emerged.

*Sub-Theme 3A (turning points)*: For the first pattern, participants noted that they reached a critical “turning point” in their lives that took various forms. For example, some reported making a subsequent suicide attempt or being admitted to an inpatient psychiatric unit. Others reported that someone close to them had attempted suicide, which led them to make a change in their own behavior. Still others reported that they eventually started to receive some form of mental health intervention (sometimes because parents or other adults found out about their NSSDV)—either psychotherapy and/or psychotropic medication—which was perceived as a significant turning point and contributor to desistance. Indeed, everyone who reported receiving therapy noted that such intervention was helpful in relieving potential desires to engage in NSSDV, either from simply talking with someone or because of coping skills learned in therapy, such as mindfulness and other cognitive-behavioral techniques. For example, one individual describes how their NSSDV eventually led to a turning point in their lives:

(ID 8): It hit a certain point where it had, it had escalated as much as it was going to escalate. I had tried to kill myself. I was institutionalized. And, I mean, you know, you try and fail to kill yourself. You either come away with it with a new lease on life, or exactly the same behavior. And I was fortunate enough to come away with it with a new lease on life kind of feeling.

*Interviewer*: So where did this thinking come from?

(ID 8): [It] came after a lot of the therapy that I had. And a lot of the interventions that worked and a lot of those things. . . But for a long time, the depression was a lot stronger than that voice.

Multiple participants commented that once their parents found out that they were engaging in NSSDV, parental involvement was instrumental in reducing or eliminating such behavior:

(ID 2): It didn’t last very long and because that’s kind of when I started going to therapy three times a week and then seeing a psychologist and my parents took the door off my hinges, so like, I never was alone. . . Yeah, it didn’t last very long.

*Sub-Theme 3B (desistence over time)*: In the second pattern of desistance, six participants reported that it was not an immediate or active process or decision. Rather, NSSDV continued—often for years—finally getting to a point where engagement in this behavior was no longer needed. This pattern was often associated with improvement in social or environmental factors (such as making more meaningful or supportive friendships). Others also discussed that they eventually became more aware of their mental health needs and learned more adaptive coping mechanisms, such as self-care or socializing:

(ID 1): I pretty much stopped doing that after I went to high school. . . ‘cause it just didn’t matter as much anymore. . . I was getting more friends. I was getting more, like, comfortable with myself. And was looking for jobs. . . And I was getting into like my older teens. . . It didn’t seem like there would be much of a point anymore. I was already diagnosed with all of those other things. So I was like, ‘ok so that makes sense.’

*Sub-Theme 3C (change in behaviors)*: Here, some individuals noted that, instead of engaging in NSSDV, they switched to engaging in other behaviors—mainly body art, such as tattoos and piercings. Notably, this activity still created a physical sensation—and had a self-expression component—but one that was more socially acceptable and less harmful.

Interviewer: Do you still engage in self-harm?

(ID 11): No. I get tattoos.

Interviewer: So, when did you stop? Or what do you think were the reasons why it stopped?

(ID 11): I know what the reasons are—my mom told me that if I can’t love myself enough to not do it, to love her enough to not do it because it breaks her heart to feel like she is losing me. And she sat down and got real with me, and turns out that was what I needed—somebody to understand.

#### Theme 4: Impulsivity and NSSDV

Fourteen individuals described their NSSDV as occurring *impulsively* (i.e., not planned or premeditated), especially when it initially started and typically within the context of strong negative emotions. As one participant noted:

(ID 12): So, another day shortly thereafter, I got into another fight with my mother because. . . I was a hormonal, going-into-puberty preteen and she was going through menopause. . . so we’re constantly butting heads. . . So, I was in my room and didn’t know what to do and in my frustration, I kinda just grabbed onto my thigh and like ripped through my skin and it was that rush again and I was like, ‘Huh! Thank god for something.’ And then, I just started kind of like obsessively scratching myself and then that kind of led to that chapter.

Similarly:

(ID 2): I feel like that was like, ‘Oh you can cut and like it makes you, makes it feel better.’ I want to say it was like something to do with that movie ‘cause, I just remember watching that movie. . . like this is, this gives you immediate relief and I think, when you get to that point, you’re like searching for anything, you know? Like. . . you’ll do anything and everything for immediate relief and not think about the long-term consequences or aspects of what you’re doing. . . It’s like an impulse, totally.

#### Theme 5: Keeping NSSDV a Secret

This theme emerged after an initial review of interview transcripts. Although many participants reported engaging in NSSDV to gain attention from others at some point during the course of their NSSDV behavior, almost half also noted that they tried to hide it from others. One, for example, noted that she cut the bottom of her feet (even to the point of not being able to walk) so that others would not notice. For another:

(ID 8): It started as like, it was a public thing that I would do when I knew someone was watching. So that I could get that initial, “Oh my god, are you ok? Oh my god what are you doing? How can I help?” Even if there was a negative response. . . It was very much that was the only thing that drove me to do it. And then as my depression got worse, it was like, maybe I can utilize this as an actual physical outlet instead of just seeking the attention. Because it went from like doing it publicly to really trying to hide it.

Most acknowledged that their NSSDV was not healthy or socially acceptable. They expressed a sense of shame or embarrassment, which was associated with a desire to keep both their feelings and their NSSDV behavior a secret. In addition, some thought that something was wrong with themselves because they believed that the NSSDV seemed helpful in the moment. This belief further drove their desire to keep it secret and deepened their sense of shame. For example:

(ID 11): No. I never even gave anybody a chance. . . I didn’t tell anybody that I was doing it, you know? I was secretive. For a child that is harming themselves and when it’s on your wrist you’re looking to get caught because somebody’s guaranteed to see it. Give them the attention. Don’t just get them counseling, but go to counseling with them, you know? Give them the first half of the session and then sit in the second half so that they can communicate with you because there’s obviously something being missed in that relationship. For me something wasn’t being missed, I was angry at myself. I had gotten myself into that situation. . . Yeah, mine was self-punishment and guilt related.

## Discussion

Self-directed violence is a significant and growing public health concern among youth. Clinical populations—especially girls with ADHD—are at particularly high risk for engaging in such behaviors (Allely, 2014; Hinshaw et al., 2012, 2022). Utilizing semi-structured, qualitative interviews, we explored multiple themes related to NSSDV, leveraging a prospective, longitudinal sample, to better understand critical individual and contextual factors affecting individuals with histories of NSSDV. In total, 57 women participated in qualitative interviews, with 24 reporting previous and/or current NSSDV behaviors (17 of whom had childhood ADHD diagnoses). Identified themes included core reasons for engaging in NSSDV, inter- and intra-personal factors associated with NSSDV, and novel findings related to the developmental course of NSSDV across childhood and adolescence. To our knowledge, this is the first qualitative study conducted with individuals with childhood ADHD regarding self-directed violence.

We identified five themes from qualitative interviews. Three of these themes are complementary to and expand upon extant theories of NSSDV, including reasons for engaging in NSSDV (Theme 1), the influence of others on NSSDV behaviors (Theme 2), and the relations between NSSDV and impulsivity (Theme 4). Regarding Theme 1 (reasons for engaging in NSSDV), findings supplement prior work, with affect regulation as the most common reason identified (Edmondson et al., 2016; Klonsky, 2007; Taylor et al., 2018), followed by the desire to gain attention from others. These findings are consistent with the functional model of NSSDV outlined by Taylor et al. (2018), with the two most common functions being emotion regulation (intra-personal) and to communicate distress (inter-personal), with fewer individuals also identifying self-punishment (intra-personal). Two inter-personal functions (interpersonal influence and punishing others) did not appear to be relevant for the present sample. Importantly, the vast majority of individuals endorsed multiple reasons for engaging in NSSDV—highlighted in several participant excerpts—suggesting the challenging, complex, and multifaceted nature of NSSDV behavior with this population.

Indeed, one of the more novel findings herein comprises the contextually sensitive stories regarding changes in the reasons for engaging in NSSDV over time. For example, some individuals noted that although they started out engaging in NSSDV to regulate affect or as a physical expression of emotional pain, they continued it in order to gain attention from others. Others noted the opposite pattern, wherein they started engaging in NSSDV to gain attention from others but then continued for affect regulation purposes. Others still noted that they engaged in the behavior primarily because they believed that they deserved pain or punishment (often because they perceived deficiencies in themselves related to their mental health or neurodiversity) or as a way of asserting control over their life – typically in the context of family and personal distress. Most, however, noted that they continued the behavior because it became habitualized.

In fact, most reported that NSSDV provided a form of temporary relief, as either (a) a positively-reinforcing sense of physiological arousal or “rush” (or through the positive reinforcement brought about from gaining the attention of others), or (b) a negatively-reinforcing “escape” from the challenges within their environment or their own distress. These findings are consistent with a framework proposed by Cummings et al. (2021), wherein individuals who have an increased sensitivity to either socioaffective pain or immediate reward might be at increased risk for NSSDV during adolescence. Individuals with ADHD, given the hypothesized neural pathways involved in the disorder, would be likely to fit into either or both of these categories (Klein et al., 2017). Indeed, many in the present study reported that their NSSDV behavior often started out impulsively (Theme 4), which is not surprising given the high prevalence of childhood-ADHD diagnoses in the present sample (see also Beauchaine et al., 2019).

One of the most salient and novel findings herein is that almost half of those who engaged in NSSDV did so in private or otherwise tried to hide their behavior from others (Theme 5). This theme might also be particularly relevant for the present female sample, given that girls with ADHD experience key problems around social competence and peer acceptance, and in some ways already feel “different” from their peers—feelings that can be exacerbated during adolescence (Kok et al., 2016). Such experiences could certainly then exacerbate feelings of self-stigma and shame. In addition, some youth describe NSSDV as being comforting in some way (Edmondson et al., 2016; Hawton et al., 2012), which could lead many to not disclose such behaviors. Regarding Theme 2, many who engaged in NSSDV noted that their behavior was at least in part influenced by others. Such social influence included TV, movies, and media—and for present-day youth, undoubtedly social media. Indeed, several individuals noted that they first saw this behavior in a movie. The high rates of peer influence may be particularly relevant for girls with ADHD given that this population is likely to experience lower social acceptance and greater peer rejection and victimization than neurotypical peers (Kok et al., 2016). As a result, some may engage in behaviors such as NSSDV to increase peer acceptance, particularly among others with like tendencies.

Regarding desistance from NSSDV (Theme 3), three patterns emerged. In the first, individuals reported never receiving intervention or treatment in any form and desisted either because of improved environmental factors or just “growing up” and not needing to engage in such behaviors any longer. This pattern is consistent with prior findings regarding a largely “adolescent-limited” nature of NSSDV (Beauchaine et al., 2019; Brager-Larsen et al., 2022; Sinclair & Green, 2005; Westers & Culyba, 2018; Wilkinson et al., 2022). A larger subgroup, however, included those whose NSSDV escalated to the point of requiring intervention, including frankly suicidal behaviors. The subsequent receipt of mental health treatment was often perceived as essential to their eventual desistance. Because adolescents may not realize that NSSDV is an indication of other mental health problems (Sinclair & Green, 2005), the importance of early screening and mental health education appears clear.

In the third pattern, a smaller number of individuals endorsed transitioning from NSSDV behaviors (e.g., cutting) to more socially acceptable forms of self-expression and physiological sensation-seeking, such as tattoos, piercings, or other forms of body art. This more novel finding has potential implications for intervention. That is, instead of asking adolescents to entirely forgo something that may have been perceived as temporarily beneficial, encouraging them to use other creative outlets or healthier means of self-expression could be considered. Clearly, this finding requires further study and replication.

The present study has several strengths, including leveraging a subsample from a large, prospective longitudinal study and the use of a qualitative interview format to obtain rich stories regarding NSSDV histories. Additionally, we believe that understanding individuals’ experiences with SDV—in their own words—can be powerful, moving, as well as informative. Limitations, however, are noteworthy. First, although the size of the subsample who reported engaging in NSSDV (25)—as well as the overall sample size (57)—was considerable for a qualitative study, it did not provide adequate power to conduct quantitative/mixed-methods analyses (e.g., exploring differences in NSSDV behavior between ADHD and comparison sub-samples). Even so, we note that the patterns of behavior (e.g., the functions of NSSDV, keeping NSSDV a secret) did appear similar across both ADHD and non-ADHD study participants. Second, several years separated when individuals engaged in NSSDV (usually in adolescence) and when they participated in qualitative interviews (mid-to-late 20s). This “lag” certainly presents a concern regarding retrospective recall. However, the concordance between the quantitative NSSDV data obtained during adolescence/emerging adulthood and the subsequent qualitative interviews was close to 100%. The key discrepancy was that three individuals did not report NSSDV during the Wave 3/4 follow-up visits but retrospectively reported NSSDV during qualitative interviews. A plausible reason could be the aforementioned feelings of shame and stigma surrounding such behaviors, especially during late adolescence and emerging adulthood. Indeed, what stands out in multiple excerpts is the level of detail and specificity participants provided as they recounted their earlier experiences. Some evidence suggests that certain life events, including highly emotional ones, may be strongly encoded and easily recalled later (Cahill et al., 1996). We add that a number of individuals reported only relatively recently realizing the interplay between their past environments, their thoughts/feelings, and their NSSDV patterns. In contrast, a smaller study that explored the narrative of six adolescents (aged 13–18) with a recent history of SDV found that participants’ overall stories about their SDV behavior were less coherent and integrated (Hill & Dallos, 2012). It may be that additional time for maturation, identity development, and introspection is be beneficial for a qualitative approach regarding SDV, despite the longer time between the behavior and interview (see also Sinclair & Green, 2005).

## Conclusion

Along with a number of quantitative empirical findings (for a review, see Hinshaw et al., 2022), the present qualitative findings underscore the risks that a childhood ADHD diagnosis incurs for NSSDV, especially in girls. For youth with ADHD or other mental health problems, feelings of shame and stigma can certainly emerge—with regard to their mental health challenges and their SDV behavior—underscoring the need for thoughtful, early education, peer support, targeted screening and intervention, as well as reducing mental health stigma. In addition, given the potential that NSSDV is influenced by others (including peers, film, media, and social media), it is also crucial that public health and policy efforts raise societal awareness more broadly on this topic. From an intervention and treatment perspective, a lack of evidence-based prevention and intervention programs specifically aimed at the treatment of NSSDV in youth is apparent, although dialectical behavior therapy is one of the most promising treatments with growing support (Asarnow et al., 2021; Beauchaine et al., 2019; Nock, 2010). Treatment, however, generally occurs after the fact; universal, programmatic, school-based mental health programs are bound to be important to aid in prevention efforts (Hawton et al., 2012; for a review, see Weare & Nind, 2011). Finally, supporting and educating parents and peers is indicated, as they are likely to play an important role in mitigating and preventing SDV (Wadman et al., 2018).

One important implication pertains to the timing of interventions. Most participants herein started to engage in NSSDV between seventh and ninth grade (see also Beauchaine et al., 2019; Wilkinson et al., 2022). Interventions starting in high school may well be too late for many. Policies and programs that support early interventions and increase resources for school-based mental health services could be crucial (Romer & McIntosh, 2005; Weare & Nind, 2011). Also, just as core treatments for children with ADHD often focus on educating and supporting parents, programs that support and educate parents regarding SDV appear indicated. Prior studies, as well as feedback from adolescents themselves, suggest that focus groups, structured group activities, informal social activities, and peer engagement could be beneficial for prevention (Coggan et al., 1997; Fortune et al., 2008; Wadman et al., 2018). Ultimately, having open, honest, and informed dialogue about SDV is crucial (Fortune et al., 2008; Westers & Culyba, 2018), and youth-targeted messaging is essential. Given that information is already being obtained by youth from TV/movies, the internet, and social media, it becomes increasingly important to carry a stronger voice from mental health providers, schools, peers, families, and loved ones.

## Supplemental Material

sj-docx-1-jad-10.1177_10870547231221729 – Supplemental material for A Qualitative Analysis of Perspectives on Self-directed Violence in a Prospective Longitudinal Study of Young Women With and Without Childhood ADHD Supplemental material, sj-docx-1-jad-10.1177_10870547231221729 for A Qualitative Analysis of Perspectives on Self-directed Violence in a Prospective Longitudinal Study of Young Women With and Without Childhood ADHD by Shaikh I. Ahmad and Stephen P. Hinshaw in Journal of Attention Disorders
