# Supplementary material for: A Qualitative Analysis of Perspectives on Self-directed Violence in a Prospective Longitudinal Study of Young Women With and Without Childhood ADHD
Source: J Atten Disord. 2024 Jan 31;28(9):1255–66. doi: 10.1177/10870547231221729 (PMC11168017; doi:10.1177/10870547231221729)
Supplement: sj-docx-1-jad-10.1177_10870547231221729 – Supplemental material for A Qualitative Analysis of Perspectives on Self-directed Violence in a Prospective Longitudinal Study of Young Women With and Without Childhood ADHD [file sj-docx-1-jad-10.1177_10870547231221729.docx]

**Supplemental Materials**

# **Section 1: Qualitative interview, self-directed violence topic – sample prompts**

- What do you think about people who might try to hurt themselves (or try to kill themselves)?
- Why do you think people may do that?
- Do you have any friends/peers/family members who tried to hurt themselves in the past?
- Have you ever harmed yourself on purpose or thought about doing that?
- Do you remember what led you to harming yourself the first time? Was there a particular event or feeling that led up to that situation?
- What about later/subsequent times?

# Do you think there was anything (or any person) that might have been able to prevent you from engaging in this behavior?

**Section 2: ADHD and self-directed violence status for interview participants**

| **ID** | **Wave 1 Diagnostic group (mean age 9.6)** | **Engaged in NSSDV** | **Made a suicide attempt** |
| --- | --- | --- | --- |
| 1 | ADHD | Yes | Yes |
| 2 | Comp | Yes | No |
| 3 | ADHD | Yes | No |
| 4 | ADHD | Yes | Yes |
| 5 | ADHD | Yes | No |
| 6 | ADHD | Yes | Yes |
| 7 | ADHD | Yes | No |
| 8 | ADHD | Yes | Yes |
| 9 | ADHD | Yes | Yes |
| 10 | ADHD | Yes | No |
| 11 | ADHD | Yes | No |
| 12 | ADHD | Yes | No |

Note: ADHD = ADHD Group, Comp = Non-ADHD/Neurotypical Comparison Group
